# Internet Pathways in Suicidality: A Review of the Evidence

**DOI:** 10.3390/ijerph8103938

**Published:** 2011-10-11

**Authors:** Tony Durkee, Gergo Hadlaczky, Michael Westerlund, Vladimir Carli

**Affiliations:** 1 The National Swedish Prevention of Suicide and Mental Ill-Health (NASP), Department of Public Health Sciences, Karolinska Institutet (KI), Stockholm SE-171 77, Sweden; E-Mails: gergo.hadlaczki@ki.se (G.H.); vladimir.carli@ki.se (V.C.); 2 Department of Journalism, Media and Communication (JMK), Stockholm University, Stockholm SE-115 93, Sweden; E-Mail: westerlund@jmk.su.se

**Keywords:** Internet use, pathological Internet use, pro-suicide websites, suicide pacts, suicide prevention

## Abstract

The general aim of this study was to review the scientific literature concerning the Internet and suicidality and to examine the different pathways by which suicidal risks and prevention efforts are facilitated through the Internet. An online literature search was conducted using the MEDLINE and Google Scholar databases. The main themes that were investigated included pathological Internet use and suicidality, pro-suicide websites, suicide pacts on the Internet, and suicide prevention via the Internet. Articles were screened based on the titles and abstracts reporting on the themes of interest. Thereafter, articles were selected based on scientific relevance of the study, and included for full text assessment. The results illustrated that specific Internet pathways increased the risk for suicidal behaviours, particularly in adolescents and young people. Several studies found significant correlations between pathological Internet use and suicidal ideation and non-suicidal self-injury. Pro-suicide websites and online suicide pacts were observed as high-risk factors for facilitating suicidal behaviours, particularly among isolated and susceptible individuals. Conversely, the evidence also showed that the Internet could be an effective tool for suicide prevention, especially for socially-isolated and vulnerable individuals, who might otherwise be unreachable. It is this paradox that accentuates the need for further research in this field.

## 1. Introduction

Suicidal behaviour is a compelling public health issue, particularly among adolescents and young adults. In Europe, it represents the second leading cause of mortality in the 10–24 year age-group [1], and among youths aged 15–29 years, the prevalence of suicide in the European region is approximately 20 per 100,000 [2]. Completed suicides among male youths account for over 66% of all suicide cases; whereas, female youths account for over 66% of all suicide attempts [3]. Research shows that for every completed suicide, there are approximately 10–40 suicide attempts [4], with some estimates reporting rates up to 100–200 times higher [5]. There is evidence showing an overall standardized prevalence of 10.5% for suicide attempts among adolescents aged 15–16 years in Europe, with large variations between countries, ranging from 4.1% to 23.5% [6]. Among youths who have previously attempted suicide, 24% attempt suicide again within the following year [7]. In addition to suicide and suicide attempts suicidal ideation (SI) and non-suicidal self-injury (NSSI) levels also appear to be elevated. Like in the case of attempts, there are considerable variations in the standardized prevalence of suicidal ideation among the general population in Europe, ranging from 1.1% to 19.8% [8]. From a global view, the estimated prevalence of suicide attempts, plans and ideation ranged from 0.4%–4.2%; 1.1%–15.6%; to 2.6%–25.4%, respectively [9]. The annual prevalence of NSSI is estimated to be around 7%, with lifetime rates between 12–13% [10]. NSSI rates appear to be higher among females than males [11].

The link between suicidal behaviour and psychopathology is relatively substantiated in the literature [12–15]; however, the connection is multifaceted. External factors that may affect the relationship between suicidal behaviour and mental health should be taken into consideration during risk assessment and prevention efforts. Among those factors which have particular relevance today, is Internet use. The Internet can serve as a channel with positive and/or negative effects on users’ psychological health and well-being. Alao *et al*. [16] and D’Hulster and Van Heeringen [17] shared this ambivalence, and suggested that the Internet can encourage suicidal behaviour by its supply of descriptions of suicide methods and pro-suicide websites, wherein individuals with severe mental health problems are advised not to seek help and, at the same time, if the Internet is used properly, it can also be a key resource for helping potentially suicidal individuals.

Referred to as the Internet paradox [18], both positive and negative effects of Internet use are observed. Observed positive effects of the Internet include the ability to utilize and disseminate information quickly and accessibly. In this context, Internet use appears very effective in a number of areas such as providing health information [19], serving as a platform for education [20], social networks and support [21], entertainment [22], and even mental health promotion and prevention programs [23]. The negative consequences of Internet use often coincide with social and risk-behavioural problems. Research shows that adolescents who are susceptible to social exclusion, bully victimization and substance abuse may utilize the Internet as a coping mechanism in an attempt to relieve stress [24]. It is under such conditions that adolescents become the most vulnerable for incipient online risks, including cyber-bullying, pathological Internet use, pro-suicide websites, facilitation of suicide pacts, and expedition of suicidal methods [25,26]. As global Internet user rates are rising, the reliance on the Internet and ensuing online risks are increasing as well.

**Figure 1:**
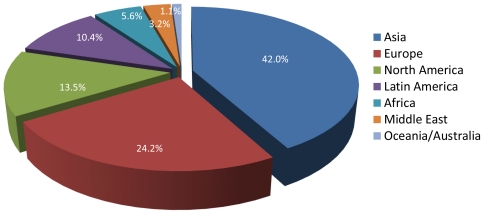
Internet users distributed by geographic region in 2010. Source: *Internet World Stats, 2010* [27].

Internet use has grown exponentially worldwide, comprising now nearly two billion users [27]. With regards to geographic distribution, in 2010 the largest number of Internet users was located within the Asian region, which accounted for 42% of global Internet users (Figure 1). Europe accounted for the second highest region, with 24.2%, followed by North America (13.5%), Latin America (10.4%), Africa (5.6%), Middle East (3.2) and Oceania/Australia (1.1) [27].

The increase in Internet usage is also illustrated predominantly among the adolescent age-groups. In EU-27, statistics demonstrate that 90% of young people aged 16–24 years used the Internet regularly during 2010 [28]. Due to the widespread use of the Internet on most continents, research concerning its implications on mental health and suicidal behaviours necessitate scientific review.

The general aim of this study was to review the scientific literature concerning the Internet and suicidality; and examine the different pathways by which suicidal risks and prevention efforts are facilitated through the Internet. The main themes that were investigated included*: pathological Internet use and suicidality, pro-suicide websites, suicide pacts on the Internet, and suicide prevention via the Internet.*

## 2. Methods Section

An online literature search was conducted using the MEDLINE and Google Scholar databases. There were no restrictions on language, time or on publication status, however, only articles published in English were found. The main identifier used in the search was *Internet* combined with co-identifiers: *suicide* or *suicide attempt* or *suicidal thoughts* or *suicidal ideation* or *suicidal behaviour* or *suicide pacts* or *pro-suicide* or *pathological use* or *addiction* or *mental health* or *prevention* or *intervention*.

The only criteria were to include studies reporting on one or more of the following main themes: pathological Internet use and suicidality, pro-suicide websites, suicide pacts on the Internet, and/or suicide prevention via the Internet. Articles were screened based on the titles and abstracts reporting on the themes of interest. Thereafter, articles were selected based on scientific relevance of the study, and included for full text assessment. An evaluation of the assessed papers was performed, and a synthesis of the results was summarized for each main theme.

## 3. Results and Discussion

### 3.1. Pathological Internet Use and Suicidality

Pathological Internet use (PIU), also referred to as Internet addiction [29], has been gaining attention in recent years, partly due to its potential inclusion in the *Diagnostic and Statistical Manual of Mental Disorders, 5th Edition* (DSM-V) nosological system [30,31]. PIU has been conceptually modelled as an impulse-control disorder [32] that shares characteristics similar to behavioural addiction: *salience, mood modification, tolerance, withdrawal symptoms, conflict and relapse* [33,34].

In an attempt to define and classify PIU, Young developed an assessment tool entitled Young’s Diagnostic Questionnaire for Internet Addiction (YDQ) [35]. The YDQ was developed according to the DSM-IV diagnostic criteria for pathological gambling. In the YDQ, the diagnosis for addiction is based on a pattern of Internet usage, over the past six months, which result in a clinical impairment or distress as indicated by the following criteria: (i) preoccupation with the Internet; (ii) need for longer amounts of time online to achieve satisfaction; (iii) repeated unsuccessful efforts to control, cut back, or stop Internet use; (iv) restlessness, moodiness, depression, or irritability when attempting to cut down or stop Internet use; (v) staying online longer than originally intended; (vi) jeopardizing or risking the loss of a significant relationship, job, or educational or career opportunity because of the Internet; (vii) lying to family members, therapists, or others to conceal the extent of involvement with the Internet; and (viii) using the Internet as a way of escaping from problems or of relieving a dysphoric mood [32,35]. The sum of the score is calculated based on the number of ‘Yes’ responses and ranges between 0–8. Those scoring 3–4 are considered ‘at-risk’ for addiction and those individuals scoring ≥5 are assumed to be pathological Internet users. The YDQ is utilized to measure pathology concerning Internet use and not only excessiveness, and has also been validated in several studies [36,37]. Despite the controversy surrounding non-standardized measures for PIU, there is research showing significantly associated risks that are remarkably consistent [38].

PIU appears to have substantial negative effects in many aspects of an individual’s well-being, such as cognition, emotion and social functioning [39,40]. Essentially, there are several cross-sectional studies conducted in this area, which suggest a potential correlation between PIU and depression [41], anxiety disorders [42], obsessive-compulsive disorders [41], and anti-social behaviours [43].

Three studies were identified in the literature that examined the direct link between PIU and self-destructive behaviours related to suicidality (*i.e.*, two on suicidal ideation [44,45] and one on non-suicidal self-injury [46]). Both studies examining PIU and suicide ideation found significant correlations, wherein pathological users had a three-to-four-fold higher risk of suicide ideation compared to non-addicted individuals. Moreover, Lam and colleagues [46] examined an adolescent sample of 1618 high school students aged 13–18 years. The authors investigated the correlation between PIU and non-suicidal self-injury (NSSI) behaviours. The results revealed that adolescents categorized as moderately and severely addicted to the Internet had a significantly increased risk of self-harm behaviours (OR: 2.0; 95% CI: 1.1–3.7) when compared with non-addicted adolescents [46].

For the reasons described above, PIU and suicidal behaviours can be considered as one of the pathways in the multifaceted risks for suicidality. Other risks include pro-suicide content disseminated on websites, which essentially jeopardizes suicide preventive efforts.

### 3.2. Pro-Suicide Websites

The Internet provides an assortment of viable websites providing endless access to information. However, is the information persons are retrieving always correct, or even safe? At present, there are a large number of pro-suicide websites in several different languages on the Internet, and they often rank high on the search engines’ results pages [47]. These often interlinking websites feature similar characteristics in offering content, wherein suicidal acts are promoted, and typically utilized as a means for individuals to cope with problems in life. In some instances, while no motivation is given, taking one’s life is encouraged as a form of rebellion against the prohibition of suicide [48]. Becker and Schmidt [49] have termed this aspect as a clear ‘anti-psychiatric’ view, which manifests itself through disseminating information on the most effective ways to commit suicide, as well as propagating that suicide should be reflected as an individual choice. On pro-suicide websites, society and its institutions are seen as a threat to the individual’s ‘natural rights’ to take their lives. These messages reach a relatively large number of vulnerable persons, with social and psychological problems, as well as those who are actually seeking help on the Internet.

The existing studies on pro-suicide websites have mainly focused on the classification of website content. This is performed by entering keywords and phrases related to suicide into a search engine. Websites ranking highest in the search are scrutinized for suicidal content and profiled as pro-suicide, suicide-neutral or anti-suicide [49–52].

Biddle and colleagues [47] conducted a study in which they examined various search engines, including Google, Yahoo!, MSN and ASK, and the outcomes each particular search engine displayed when entering select keyword and phrases, e.g., (*a*) suicide; (*b*) suicide methods; (*c*) suicide sure methods; (*d*) most effective methods of suicide, *etc.* [47]. Subsequently, as users seldom look beyond the first ten hits on the results page, the study outcomes were thus confined to this criterion. This generated a total of 480 results. The study indicated that nearly 30% of the content of web pages was subjugated by material concerning suicide methods. Suicidal acts ranged from incitement, provocation to non-rejection. Conversely, 25% of the webpage content focused on suicide prevention, with a distinctive opposition to suicidal behaviours, while 40% were construed as embracing, to some extent, a position of suicide prevention [47,48].

Recupero and colleagues [53] investigated the accessibility of harmful online resources for suicidal persons. The authors utilized five popular search engines: Google, Yahoo!, ASK, Lycos and Dogpile. The authors entered four suicide-related search terms: (*a*) suicide; (*b*) how to commit suicide; (*c*) suicide methods; and (*d*) how to kill yourself. Search outcomes were categorized as *pro-suicide, anti-suicide, suicide-neutral, not a suicide site,* or *error* (*i.e.*, page would not load). Results showed 373 web pages comprising suicidal content. Among those web pages, 11% were considered to be contain obvious pro-suicide material; 30% was deemed to be suicide-neutral, and 29% was anti-suicide [48,53].

It is important to note that pro-suicide communication on the Internet seems to have become more common over time. A study using the Google search engine to investigate the prevalence of suiciderelated material on the Internet showed that there was a proportional increase in pro-suicide messages and discussions between 2005 and 2009 [54].

In the 2009 study *Internet monitoring of suicide risk in the population*, McCarthy [55] utilized a service provided by Google, entitled Google Trends. This service provides search volumes, *i.e.*, data about what information users have searched for over time. McCarthy compared the search volumes using specific search terms as: (*a*) suicide, (*b*) teen suicide, (*c*) depression, (*d*) divorce and (*e*) unemployment with statistics on suicide and NSSI from the US Center for Disease Control (CDC). The results showed that the Google search volumes correlated with both suicide and NSSI; however, differed among age-groups [55]. The population, as a whole, displayed an overall negative correlation, *i.e.*, in periods of higher search volumes for the above-mentioned keywords, there were a decrease in suicides and self-harm acts. According to the author, this could be explained by the fact that most persons use the Internet for help-seeking reasons. In contrast to the general population, there was a positive correlation for young people between 15–25 years, *i.e.*, in periods of high search volumes; there were an increase in suicides and self-harm acts in this group. This indicates that young people, to a higher extent than the general population, use the Internet for self-destructive purposes. The study supports the postulation of a significant correlation between search activity on the Internet and suicide. Furthermore, the results suggest that Internet searches on suicide-related terms can be a predictor of suicide and NSSI.

Pro-suicide websites are produced in opposition to socially dominant attitudes on the topic of suicide [56]. In our society, the issue remains to question the primacy or sanctity of life. To advocate the individual’s ‘right’ to end their life is the primary argument from the pro-suicide view. It is an effective weapon in the struggle against society’s established morals and values. Descriptions of suicide on pro-suicide websites are, thus, tools for distinguishing the self and the group from the worldview of the dominant culture. Although construction of the pro-suicide approach can, in many ways, reflects a destructive activity, it also constitutes a meaningful activity for its protagonists. The reasons why pro-suicide content is produced and available for public access is perhaps conceived as a meaningful identity-constructive role it fulfils for the producers.

Baker and Fortune [57] argued that discussions in various studies and media have been too generalized, lacking in-depth knowledge concerning Internet communication on suicide and self-harm, and its insinuation for those involved. Based on ten in-depth interviews, with people who regularly visited self-injury and suicide forums, the authors concluded that these forums provided participants a source of empathy, fellowship and coping with social and psychological problems.

Westerlund [58] noted that visitors on interactive suicide forums are provided with an opportunity to discuss difficult experiences, which would not be possible in most other contexts. Participants are not held accountable to institutional figures or regulations. Their discussions, however, can potentially be destructive, wherein information concerning potent suicide methods are discussed and exchanged, and participants exhort one another to follow through with their suicidal plans. The atmosphere can also be aggressive, with elements of verbal insults and bullying, possibly having a negative impact on individuals who already feel exposed and vulnerable. At the same time, a comforting, supportive and understanding attitude can be found in many exchanges. There is an opportunity to meet other people who share similar experiences, wherein their thoughts and feelings are not condemned nor lectured about. Supportive and consoling discussions, composited with aggressive and destructive elements, become a flow of polyphonic voices. In view of this ambiguity, it is important to take a balanced view and avoid focusing solely on the potential risks inherent in chat rooms such as these.

Notwithstanding, pro-suicide websites are detrimental among a subgroup of persons with mental health problems, especially if the person is susceptible to social isolation and lacks a social network to counterbalance the negative information they may receive from such harmful websites. Pro-suicide websites often provide an open forum to discuss methods and plans for committing the suicidal act itself. Under these types of settings and circumstances is where suicidal pacts can emerge.

### 3.3. Suicide Pacts on the Internet: “Net Suicides”

The definition of a suicide pact is a cooperative choice by two or more individuals who agree that both, or all, will commit suicide together, in a prearranged place and at a designated time [59]. Research concerning the Internet and suicidal behaviour, in this aspect, investigates what is known as “net suicides”, *i.e.*, suicide pacts made on the Internet [60].

Evidence shows that suicide pacts often develop in chat rooms or on message boards, which endorse suicidal behaviours [49]. This milieu attracts vulnerable persons feeling socially excluded from society [60]. The socially-isolated individual can communicate interactively and anonymously, thus, exposing him/herself to impending pro-suicidal incitement from one or more parties involved. This could potentially lead to the coordination of a suicide pact.

An example of such a tragedy occurred in Japan during 2004 when nine people took their own lives, in a suicide pact, initiated and coordinated over the Internet [60]. According to Naito [61], in Japan alone some 60 persons a year are presumed to have died from ‘net suicides’ and these trends appear to be on the increase. However, this occurrence is not only limited to Japan. Online suicide pacts have been reported in other nations as well, including the United Kingdom, Norway and South Korea [48].

Destructive communication through Internet websites has augmented the suicidal risks for vulnerable individuals. In some cases, persons are coerced into consenting to a suicide pact by unknown accomplices. In other cases, acquaintances or friends decide collectively to formulate a suicide pact. Notwithstanding, this phenomenon may explicate the spread of new suicide methods across continents [62]. In Japan, during 2008, there was a sudden increase in hydrogen sulphide poisoning, which developed as a potential method for committing suicide. This was eventually linked to a website that disseminated material on a new technique for manufacturing gas, which was then transmitted through message boards on the Internet. As the trend rapidly spread through Internet communication, the new suicide method began emerging in other parts of the world [63].

In contrast to the potential risks of cyber technology, there is a clear advantage to having the ability to quickly access information and to interactively communicate with individuals in real-time, while maintaining anonymity. This Internet pathway has significant potential for promoting prevention efforts and reaching vulnerable risk-groups.

### 3.4. Suicide Prevention via the Internet

The prevention of suicide and suicidal behaviour is an important public health concern, yet, the topic of suicide is still subjected to stigmatization [64,65]. Suicidal behaviour has shown to significantly correlate with multiple psychopathologies, such as: depression [66,67], schizophrenia [68], anxiety [69,70], impulsivity [71], social phobias [72], obsessive-compulsive [73] and affective disorders [74]. Adolescents and young people often share comorbid risk-factors associated with suicidality, which are not always a diagnosable disorder, rather are often presented through risk-taking behaviours. There is substantial evidence showing a strong correlation between suicidality and different forms of risk-behaviours, including: NSSI [75], substance abuse [76], tobacco use [77], delinquency [78], aggression [79], bullying [80–82], and promiscuous sexual behaviour [83]. These groups are the most susceptible for incipient suicidal behaviours. Therefore, this group would be the ones who would probably benefit the most from receiving anonymous treatment online, wherein they can openly discuss their feelings without being exposed to the stigmatization and taboo of discussing mental health issues and suicidality. The ability to remain anonymous in a conversational community increases the willingness to confess and discuss thoughts and feelings related to suicide, mental pain and vulnerability, while reducing the risk of self-censorship [54]. Given the uniqueness of this specific risk-group, the Internet can prove invaluable in reaching those individuals, who otherwise are unattainable, in order to promote mental health and prevent suicidal behaviours.

Effective prevention strategies targeting this particular risk-group should include components that increase awareness and help-seeking behaviours, while decreasing risk-taking and suicidal behaviours, thereby, reducing stigma [84–87]. Wasserman and Durkee [88] have delineated specific approaches often utilized in suicide preventive interventions, among which, includes the Universal/Selective/Indicated (USI) model. In the USI model, the universal intervention targets the general population, the selective intervention targets subgroups at-risk for suicide, and the indicated intervention is aimed at high-risk suicidal individuals who already begun self-destructive behaviours [89]. The USI model that adopts the conceptual framework of increasing awareness, education and de-stigmatizing mechanisms would be a theoretical basis in developing an effective Internet-based prevention program.

Literature suggests that web-based communication can provide support to suicidal individuals. In one study, performed over a 11-month period, the results illustrated that the discussion members on a website, converging on the theme of suicide, provided a supportive network, based on shared experiences, sympathy, acceptance and encouragement [48].

In another study, a web-based intervention on treatment-seeking among college students at-risk for suicidality was examined. Participants from two universities were invited to complete an online survey that screened for depression and other suicidal risk-factors. Respondents received a modified and personal assessment and were able to converse anonymously with an online clinical therapist. Students classified as at-risk were advised to appear in-person for evaluation and treatment. Results yielded 1162 students, in which 8% of invitees completed the screening questionnaire; 981 (84.4%) were considered to be at high- or moderate-risk. Among this cluster, 190 (19.4%) joined a personal examination session, and 132 (13.5%) entered therapy. Outcomes stipulated that students who engaged in online discussions, with the clinical therapist, were three-times more likely than the in-person attendees to come for evaluation and enter treatment [90].

Although research on Internet-based suicide prevention is still fairly limited, there are examples of promising suicide preventive interventions performed over the Internet [91,92]. There are many advantages in using the Internet for prevention efforts; however, the theoretical concepts must be developed pragmatically.

## 4. Conclusions

The Internet facilitates social and psychological interaction between individuals and enables easy access to a broad array of information. This development has both positive and negative consequences for suicidality. Given that adolescents constitute the majority of Internet users worldwide, they are more likely to be influenced by the Internet. Individuals suffering from mental illness are already predisposed to the dangers that lurk on the Internet, either on the basis of their psychological disorder, or due to social isolation and/or limited social networks. On the other hand, they are also inclined to receive the help that the Internet can provide. It is this paradox that encompasses research on Internet pathways in suicidality.

Overall, the results of this review have illustrated specific Internet pathways that increase the risk for suicidal behaviours, particularly in adolescents and young people. Several studies have found significant correlations between PIU, suicidal ideation and NSSI. Pro-suicide websites and online suicide pacts were observed as high-risk factors for facilitating suicidal behaviours, particularly among susceptible individuals. Inversely, the evidence showed that the Internet could also be an effective tool for suicide prevention, especially for socially-isolated and vulnerable individuals, who otherwise were unattainable. This provides a basis to spread awareness, education and support required to promote mental health and prevent suicidal behaviours.

Through the Internet, the preventive understanding of suicide is challenged by new voices, and the battle over definitions of “right and wrong” and perceptions of “true and false” has intensified. The possibility of reaching out to large groups of users is no longer monopolized by institutional senders. These developments brought on by the wide spread use of the Internet have posed obstacles, as well as benefits, in the field of suicide prevention.
